# Construction and Demolition Waste as Recycled Aggregates in Alkali-Activated Concretes

**DOI:** 10.3390/ma12234016

**Published:** 2019-12-03

**Authors:** Zahra Abdollahnejad, Mohammad Mastali, Mahroo Falah, Tero Luukkonen, Mehran Mazari, Mirja Illikainen

**Affiliations:** 1 Civil and Environmental Engineering Department, University of Connecticut, 261 Glenbrook Road Unit 3037, Storrs, CT 06269-3037, USA; 2 Fibre and Particle Engineering, Faculty of Technology, University of Oulu, P.O. Box 4300, 90014 Oulu, Finland; muhammad.mastali@gmail.com (M.M.);; 3 Department of Civil Engineering, California State University, Los Angeles, CA 90032, USA

**Keywords:** alkali-activated concrete, recycled aggregate, construction and demolition waste, sustainability

## Abstract

The growth of global construction has contributed to an inevitable increase in the amount of construction and demolition (C&D) waste, and the recycling of C&D waste as aggregates in concrete is receiving increased interest, resulting in less demand for normal aggregates and bringing a potential solution for the landfilling of wastes. Recently, several studies have focused on the use of C&D waste in alkali-activated concrete to move one step closer to sustainable concretes. This paper focuses on the main mechanisms of using C&D waste in the resulting physical, mechanical, and durability properties of alkali-activated concrete in fresh and hardened state properties. The main difficulties observed with recycled aggregates (RA) in concrete, such as high levels of water demand, porous structure, and low mechanical strength, occur in RA alkali-activated concretes. These are associated with the highly porous nature and defects of RA. However, the high calcium concentration of RA affects the binder gel products, accelerates the hardening rate of the concrete, and reduces the flowability of alkali-activated concretes. For this reason, several techniques have been investigated for modifying the water content and workability of the fresh matrix and for treating RA and RA/alkali-activated binder interactions to produce more sustainable alkali-activated concretes.

## 1. Introduction

There is an increasing global demand for aggregates in the concrete industry. It is estimated that the global demand for aggregate will rise at an annual rate of about 5.2% and reach 51.79 billion metric tons in 2019 [1]. The excessive use of natural aggregates (NA) has raised a growing concern about the depletion of resources and environmental degradation. To tackle this challenge, the use of recycled aggregates (RA) has received significant attention.

One of the main sources of RA is construction and demolition (C&D) waste. Based on European legislation of waste management, approximately 850 million tons of C&D waste is generated in the European Union each year, which represents 31% of the total generated waste [2]. However, current estimates show that only 3% of the overall aggregate consumption originates from RA [1]. Based on these estimates, the reuse of this quantity of waste material provides an opportunity to move towards greater sustainability through partial or total replacement of NA in the production of concrete.

C&D waste can contain different components, including crushed old concrete from existing concrete structures (either ordinary Portland cement [OPC] or non-OPC based), bricks and stones, fragments from crushed masonry elements, different types of ceramics (such as tiles and sanitary ceramics), glasses, and more. This random matrix distribution causes some limitations in the use of these materials with regard to standards for the maximum volume of RA in concrete. For instance, European standards recommend no more than 30 wt % RA in high-strength concrete [3,4]. Several investigations have been carried out on using C&D wastes as RA in cementitious composites. So far, OPC is the main binder used to produce concretes with RA. However, the high rates of production of OPC has been challenged due to its high energy consumption during the calcination process, excessive quantities of greenhouse gas (GHG) emissions, depletion of natural resources (in 2017, cement production was 4150 (Mt), and global fossil energy production was around 480 EJ/year [5]), and the generation of dust. These factors have resulted in a high demand for alternative approaches to sustainable concretes [6].

Alkali-activated concretes with various availabilities, reactions, costs, and CO_2_ emissions in production have been proposed as alternatives to OPC-based concretes and have shown acceptable mechanical and durability properties as well as lower environmental impacts [7]. Alkali-activated concretes are commonly comprised of aluminosilicate precursors such as fly ash, volcanic ash, metakaolin; alkali activators such as sodium hydroxide, and sodium silicate; and aggregates [8,9,10,11]. The recent approaches to develop these sustainable concretes have been through the incorporation of recycled materials and minimizing the need for raw materials. Recently, several studies have investigated the potential for using alkali-activated concretes and RA to produce green concretes. This review surveys the mechanisms and factors that influence the properties of fresh and hardened alkali-activated concretes made with RA recovered from C&D waste. In addition, problems involved in using RA and possible solutions to minimize the negative impacts of its use are also deliberated.

## 2. RA in Alkali-Activated Concretes

**Figure 1:**
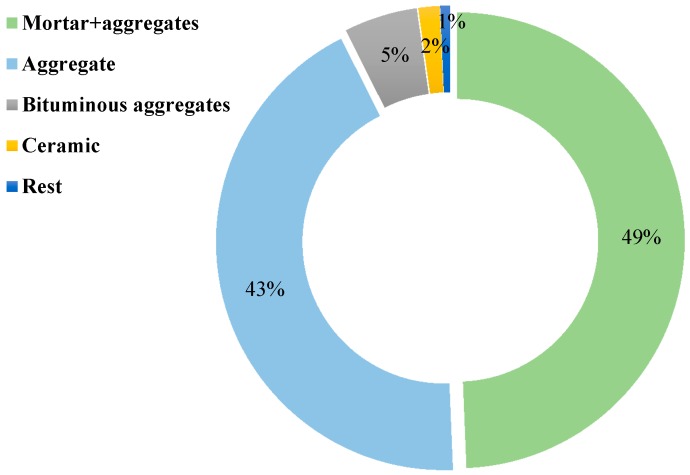
The composition of C&D waste [15].

**Figure 2:**
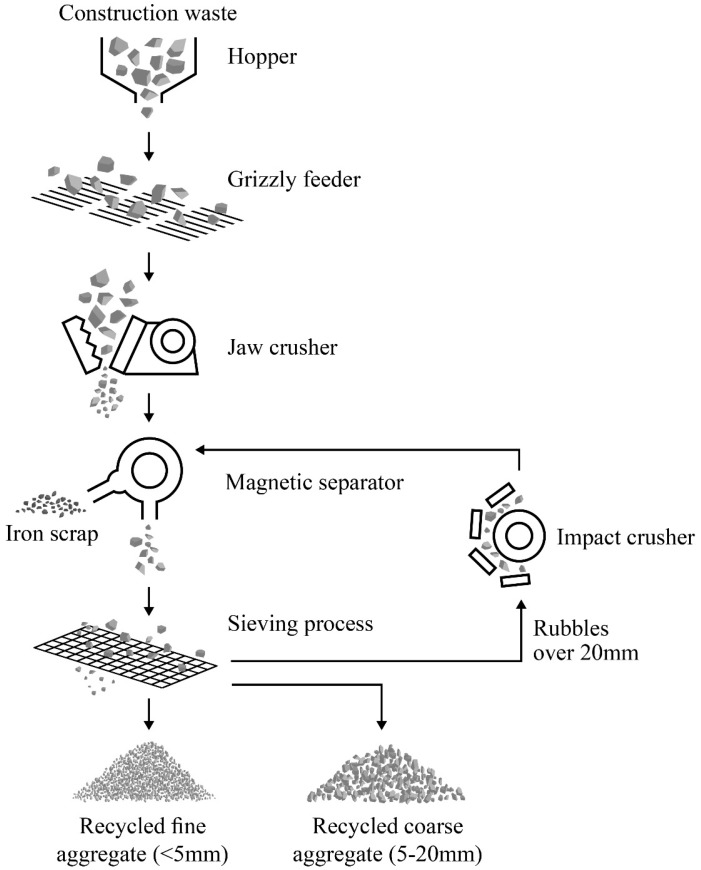
Recycling process of C&D waste to produce RA [13].

Figure 1 displays an estimated RA composition obtained with respect to EN 933–11 [12]. Several recycling processes should be implemented with the preparation of C&D waste to use it as RA. As indicated in Figure 2, the recycling processes include initial inspection, crushing, magnetic separation, and vibrating screens [13]. Following this procedure, the product is mainly NA adhered with cementitious mortar or fragments of hydrated binder. However, there are sources of impurities and contaminants that impact the physical and mechanical properties of RA [14].

### 2.1. Fresh State Properties

The incorporation of C&D waste as RA in alkali-activated concretes reduces the flowability and accelerates the setting time for the following reasons:

- C&D waste is porous and absorbs the liquid phases of the system.
- C&D waste is a source of reactive precursors, such as Ca.

**Figure 3:**
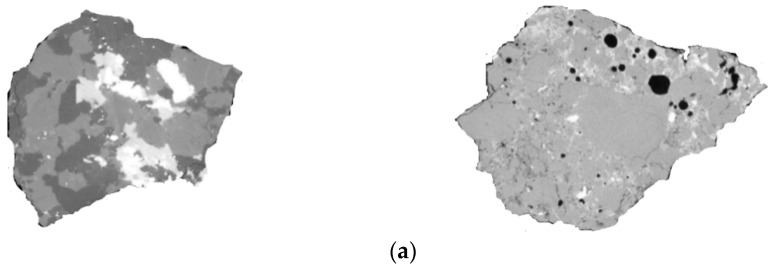

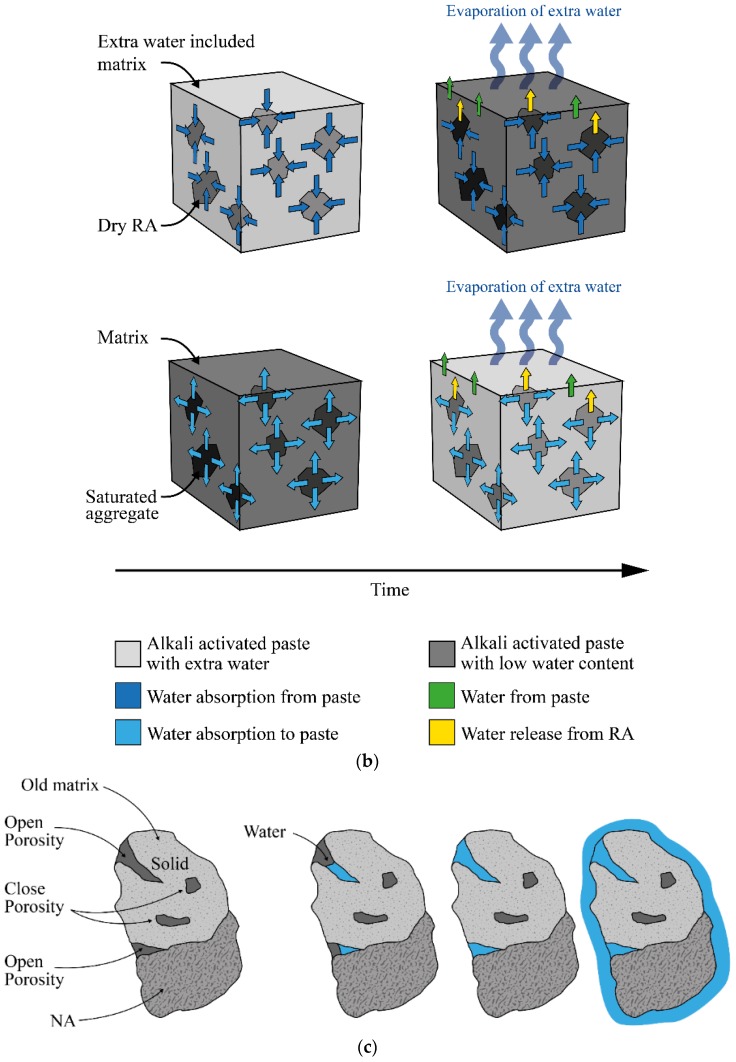
(**a**) 3D scanner analysis of NA (left side) and RA (right side) [16]; (**b**) Mechanism of adding additional water during the mixing procedure and using pre-saturated RA [34]; (**c**) Reduction of porosity of RA using pre-wetting [35].

The main problem with using C&D waste as RA in the fresh state properties of cementitious materials is the high level of water absorption that significantly affects the setting time and flowability. This is due to the extremely porous structure of RA, as observed in Figure 3a [16]. The bulk density of RA without any treatment varies in a range of 1200 to 1500 kg/m^3^ [17], while this range for NA is 1500 to 1700 kg/m^3^ [18]. It has been shown that fully replacing NA with RA increases the porosity of the matrix to about 3.5–6.5% [17,19]. In the same way, RA has a higher rate of water absorption (3–13%) when compared to NA (0.5–1%) [17,20,21].

As depicted in Figure 3b, two techniques have been proposed to minimize the absorption rates of RA, including (1) pre-saturation, and (2) adding additional water during the mixing procedure [22]. Adding additional water during the mixing procedure could have better performance in terms of compensating for the high level of water absorption than a pre-saturation approach. The additional water during the mixing procedure leads to reduced pH values, activator dilution, and compensation for the high level of water absorption in RA. These parameters could significantly affect the fresh and hardened state properties of the compositions; therefore, the content of the added water becomes crucial. As shown in Figure 3c, using pre-saturated RA results in decreased porosity of the RA. Moreover, as illustrated in Figure 3b, releasing water from the pre-saturated RA into the matrix could have two additional inverse impacts: (1) it could reduce the pH values and activator dilution, and (2) this mechanism results in internal curing.

Using pre-saturated coarse RA led to a decrease in the air voids, while replacing 30–100 wt % of pre-saturated coarse RA with pre-saturated fine RA increased air voids by 40–250% [23,24]. Conversely, Toumas et al. [25] reported that using 100 wt % pre-saturated coarse RA in the cementitious composition increased the total porosity of the composition up to two times when compared to the use of dry RA. This result has been associated with the impacts of the saturated aggregates on the porosity of the new mortar [25,26]. In addition, the presence of additional absorbed water inside the RA has diluted the alkali solution [27]. Nuaklong et al. [28] showed that increasing the concentration of sodium hydroxide from 8 M to 16 M in fly ash-based geopolymers consistently reduced the flow slump diameter for both recycled and NA. Increasing the sodium hydroxide molarity increased the pH of the system and, therefore, accelerated the dissolution of reactive silicates and aluminates, which increases the viscosity and reduces the flowability of binders. A comparative study was executed to clarify the effects on the flowability of fly ash-based geopolymers containing NA or RA when changing the ratios of sodium silicate to sodium hydroxide from 0.5 to 1.5. The results showed that increasing the ratio of sodium silicate to sodium hydroxide consistently reduced the slump flow diameter in all mixtures. Moreover, as was expected, considering the same RA content, it was found that an increase in the ratio of alkali solution to the binder increased the slump flow diameter [29]. In addition to the main component of geopolymers, using chemical admixtures or superplasticizers could partially control the abrupt drop in the slump. Parthiban and Saravana [30] showed that using a 2% superplasticizer in alkali-activated slag binders containing 100 wt % RA led to limiting the slump drop to not more than 12% compared to NA. The same approach was used in OPC-based matrices, and water-reducing admixtures (<2% or 0.01 cement mass) was used to decrease the water requirement by up to 10% [22,31]. Using both treatment techniques for RA (pre-saturation and water-compensating methods) provides the mixtures with predictable workability; however, it increases the cumulative bleeding [22]. These would be more challenging for alkali-activated materials, where releasing the excess calcium content of the RA influences the setting time of alkali-activated materials [10]. In a low-calcium system, sodium aluminosilicate hydrate (N-A-S-H) gel is mainly formed, while in high-calcium alkali-activated systems, both N-A-S-H and calcium aluminate silicate hydrate (C-A-S-H) gels are formed, and the setting time depends on the dominant gel. The formation of C-A-S-H gel shortens the setting time, while the formation of N-A-S-H gel prolongs the setting time [32]. A high calcium content provides extra nucleation sites for precipitation of dissolved species, which increases the solidification rate and causes rapid hardening [33].

### 2.2. Mechanical Characterizations and Their Influential Parameters

#### 2.2.1. Mechanical Properties

**Figure 4:**
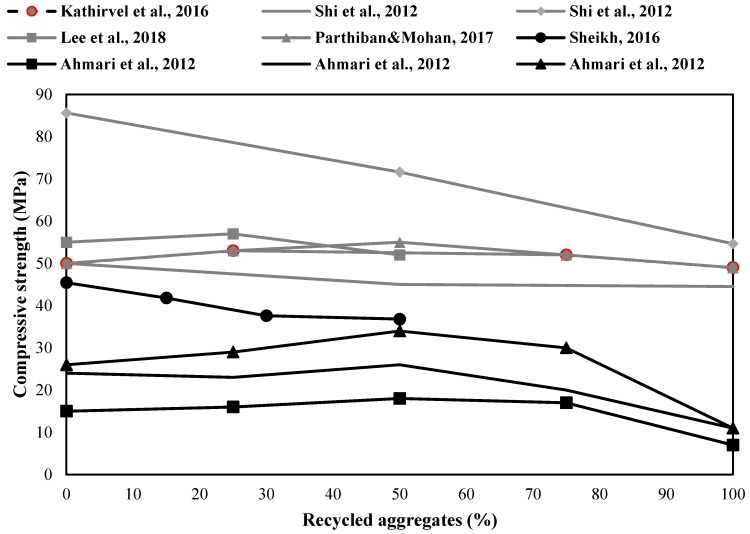
Effects of different RA content on the compressive strength in alkali-activated concretes (black lines: fly ash as the binder; gray line: slag as the binder) [30,36,37,38,39,69].

In general, replacing NA with RA degrades the mechanical properties of alkali-activated compositions [30,36,37,38,39,40]. Figure 4 shows that replacing NA with RA in different alkali-activated concretes up to 50 wt % had less impact on strength reduction (lower than 20%), while full replacement of NA reduced the final strength more than 50%.

A reduction in the compressive strength of up to 30% has often been reported when NA is fully replaced with RA in cementitious compositions [41,42,43,44,45,46,47,48,49,50]. However, using a slightly larger quantity of cement (~5%) in cementitious compositions containing RA (fully replacement) resulted in a similar compressive strength and workability as the corresponding quantity of NA [2].

There are three main concerns with the addition of RA in alkali-activated concretes, as well as in other cementitious materials, as follows:

- RA results in low strength due to a porous and cracked structure [51].
- The extra water used in RA reduces the dissolution rate of the aluminosilicate precursors of an alkali-activated matrix [37].
- There is a weakness in the interfacial transition zone (ITZ) [52].

However, the unhydrated cement portion of RA (recovered from old OPC concrete), as a source of Ca, stimulated the chemical reactions of alkali-activated systems and densified the interface microstructures [37,53,54]. This improvement has been attributed to the additional formation of calcium silicate hydrate (C-S-H) gel from the calcium present in RA. In this section, these mechanisms are studied in more detail.

***Porous and cracked nature of RA***

The porous and cracked structure of RA interferes with the mechanical properties of the corresponding concrete [55]. These properties are associated with the properties of old binders and crushing manufacturing [35]. It has been noted that when a semi-brittle material is subjected to either a mechanical or environmental load, internal stresses will be more critical around the pores where the effective cross-section is smaller [56,57]. In this regard, several experiments report that the replacement of NA with RA reduces the elastic modulus of alkali-activated concretes by up to 50% [36,58,59,60,61]. In order to minimize the negative influences of RA substitution for NA on the mechanical properties of concrete, some studies have limited the replacement to 25 to 40 wt % [62,63,64,65,66,67].

***Weak ITZ***

**Figure 5:**
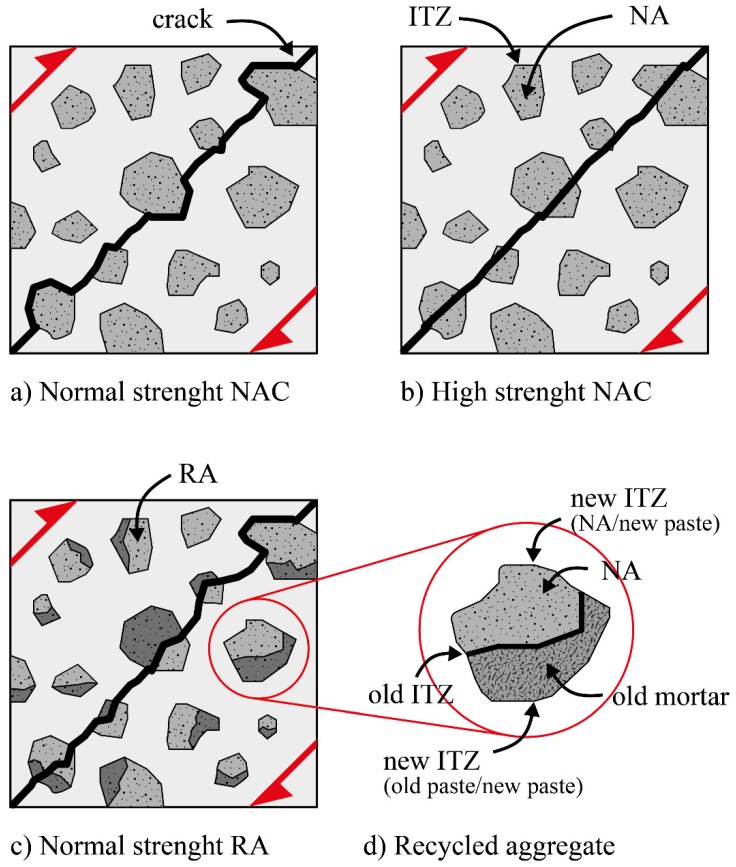
Crack propagation in compositions containing: (**a**) NA with normal strength, (**b**) NA with high strength, (**c**) RA with normal strength, and (**d**) different ITZs in the RA composition [68].

In general, when a crack meets an aggregate, it either passes through the aggregates or propagates at the aggregate/binder ITZ, depend on which one is weaker. As illustrated in Figure 5, in high-strength concrete, cracks propagate through NAs due to excellent bond properties between matrix and aggregate at the ITZ, while it propagates at the ITZ zone in low-strength concretes [68]. This scenario would be more complex in concrete incorporating RA. There are four possible areas that would have an effect, three of which involve the bond properties at the ITZ: (1) the strength of the NA; (2) the bond properties of the old mortar and NA at the ITZ; (3) the bond properties of the old mortar and the new matrix at the ITZ; and (4) the bond properties of new matrix and NA at the ITZ [64]. Kathirvel and Saravana [69] used pre-wetted and saturated RA prepared from old OPC concretes in steel-reinforced sodium silicate, and hydroxide-activated blast furnace slag concretes. They reported that paste filled the pores of the RA and resulted in an improvement of the ITZ microstructure [69,70]. The improvement at the ITZ had greater effects on the splitting tensile strength than on the compressive strength [35]. Xie et al. [71] and Koushkbaghi et al. [72] reported that the denser matrix in alkali-activated concretes and increasing sodium silicate to sodium hydroxide ratio can modify and improve the bond properties at ITZ between matrix and RA, respectively.

In alkali-activated concretes containing RA, a denser and stronger ITZ can form between aggregates and the matrix due partially to the contribution of the unreacted calcium content of attached cementitious mortars to the RA and the formation of additional C-A-S-H gels, which modified the microstructure of the matrix where the gel acted as a micro-aggregate to fill the gaps [28,73]. On the other hand, Xie et al. [74] declared that unreacted calcium content of attached cementitious mortars to the RA in ground-granulated blast-furnace slag (GGBFS)/metakaolin based geopolymers carbonates and provides the crystalline of calcite (CaCO_3_).

***Extra water in RA incorporated in alkali-activated concretes***

The high levels of water absorption of RA decrease the mechanical strength of alkali-activated concretes. RA absorbs more free liquid solutions than NA, leading to less residual alkaline solution in the mixture and, therefore, a lower geopolymerization. In other words, it reduces the activator to binder ratio. Navarro et al. [74] showed that using RA led to significant strength loss due to the absorption of activating solution in alkali-activated ground SiMn slag mortars. In addition, if the RA is pre-saturated to enhance the workability, the higher water content in the matrix will dilute the alkali activator, which results in a lower pH environment [75]. Similarly, Nuaklong et al. [28] showed that the tensile strength of high-calcium fly ash-based alkali-activated binders with sodium hydroxide 8 M was lower than with compositions containing NA (more than 15%), but increasing the molarity to 12 M and 16 M resulted in an equal strength or greater strength (up to 14% increase), respectively. Furthermore, decreasing the molarity of sodium hydroxide (16 M to 8 M) in alkali-activated concretes containing RA decreased the flexural strength [28]. In the same way, in fly ash-based geopolymers, Arenas et al. [76] used two different coarse aggregates, crushed granite, and RA activated by two different molar ratios (Na_2_O/SiO_2_), 0.29 and 0.98. The compressive strength of Na_2_O/SiO_2_:0.29 was lower than that of Na_2_O/SiO_2_:0.98 due to a lower rate of aluminosilicate dissolution. However, increasing the concentration of the alkali activator would not necessarily enhance strength because excess alkali OH^-^ ions lead to aluminosilicate product precipitation at an early stage, resulting in a decrease in strength [33,77]. Along these same lines, Mastali et al. [78] showed that increasing the concentration of sodium hydroxide from 7 M to 8 M increased the flexural strength (up to two times), while the strength was reduced beyond this concentration (up to 45%). This reduction was explained by an increase in the coagulation of silica due to a high concentration of sodium hydroxide. In addition to mechanical strength, variation in the concentration of the alkali activator greatly affects the ultimate drying shrinkage [78].

**Figure 6:**
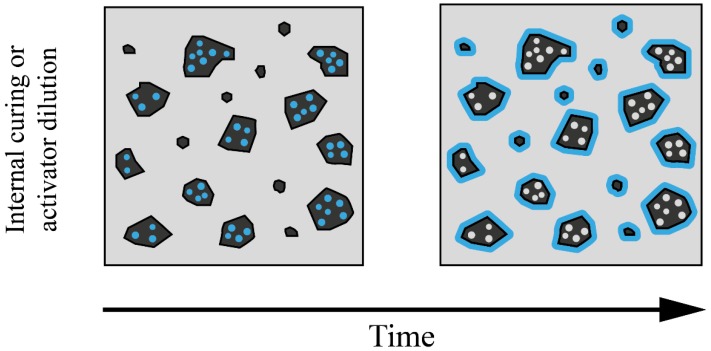
Impacts of releasing water from the saturated RA to the matrix [83].

Using RA with a high water content demand increases the drying shrinkage of concretes containing RA [35] by up to 50–70%, depending on the replacement level of RA that is associated with the high levels of water demand [79,80,81]. Greater shrinkage has been reported in alkali-activated fly ash based binders containing RA, reaching approximately 50% of the total drying shrinkage rate in the first seven days [78]. However, RA has also been employed as an internal curing agent in alkali-activated slag-based concrete (see Figure 6), which resulted in the reduction of autogenous and drying shrinkages [37]. The use of RA with a diameter greater than 9.5 mm resulted in a lower level of water absorption (3–6%), while using aggregates with diameters of 2–9.5 mm and water absorption of 8.5% showed better performance in terms of water reservoir. Using RA with a diameter less than 2 mm provided the best internal curing conditions in alkali-activated slag binders because it reduced the water supply distance from the agent to the matrix [37,82].

#### 2.2.2. Treatment of RA

**Figure 7:**
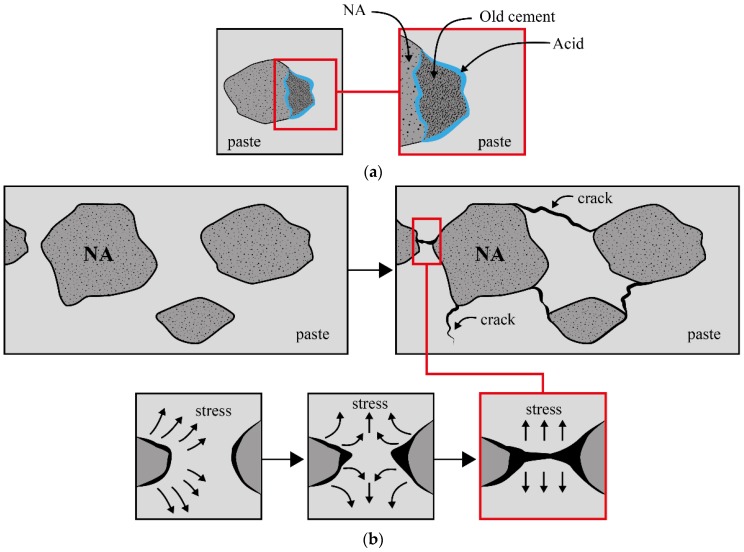
A schematic view presenting the general mechanism of removal of the adhered mortar from the aggregates by (**a**) using an acidic solution and (**b**) generating external stresses.

Regardless of the replacement content, the effects of RA in concrete depend on the quality and surface characteristics of the RA [79,84]. For instance, RA recovered from high-strength concrete produces stronger concrete than RA derived from normal-strength concrete [35]. In order to minimize the negative impacts of using RA, a variety of processing techniques have been adopted, such as pre-saturation treatment [85,86], an ultrasonic cleaning method [17], carbon dioxide sequestration [78,87], a nitric acid dissolution method [86], the freeze/thaw method [88], the thermal expansion method [89], the microwave heating method [90], the heating and rubbing method [91,92,93], and the mechanical grinding method [94]. The main concept behind these treatment methods is the removal of the adhered mortar from the aggregates by either dissolving it in a solvent or by applying internal stress at their ITZ to cause them to separate (see Figure 7). For example, the nitric acid dissolution method involves immersing the RA in a 20% (by volume) nitric acid solution and heating it until the adhered mortar starts to dissolve (approximately two hours), leaving behind the original aggregate [86]. With the freeze/thaw approach, two different steps are used to treat the RA, including the creation of mechanical stresses by subjecting the RA to a freeze and thaw action; then chemical degradation is employed by using a sodium sulfate solution [88]. The thermal expansion method consists of several cycles of soaking the aggregate in water and then heating it [89]. The microwave heating method takes advantage of the difference in the electromagnetic properties of the adhering mortar and the NA to generate high thermal stresses within the mortar, especially at the interface with the embedded NA, to cause delamination. The generated stresses also result in lumps of mortar breaking into smaller pieces [90].

The mechanical grinding method is a popular and simple treatment approach in which ball and crushing are used to remove the adhered mortar from aggregates. However, it is possible to have some damage in the obtained aggregates due to the mechanical grinding of RA [94]. However, the removal of old cement is often difficult.

One of the more effective solutions for improving the quality of RA is carbon dioxide sequestration through the densification of the microstructure of carbonated recycle aggregates or through the decomposition of C-S-H gel [78,87]. Li et al. [95] reported that there was an improvement in compressive strength (≈20%) and elastic modulus (<8%) due to the use of carbon dioxide sequestration on the hydration products (formation of calcium carbonate or calcite) and their precipitation in the pore spaces, which can improve mechanical properties by forming a denser microstructure. Mastali et al. [96] used simultaneous carbonated RA (4.2% CO_2_, 40% RH, at 20 °C for 48 hours prior to mixing components) and a flow-through CO_2_ curing method after casting in fly ash-based alkali-activated binders. They found that using both significantly increased the compressive strength up to nine times more than RA and curing at ambient temperature. The microscopic analysis of the mixtures showed that the morphology of the mixtures was changed, and more stable polymorphs were formed [96].

#### 2.2.3. Curing Conditions

It is well known that hot curing increases the mechanical strength of alkali-activated binders [97]. This finding causes from forming a harder matrix and better bond properties at ITZ between RA and matrix when compared to the ambient conditions. Robayo-Salazar showed a superior compressive strength of alkali-activated concrete with RA when it was cured at 70 °C rather than at 25 °C [58]. A similar result was obtained by Posi et al. [98] in lightweight geopolymer concrete containing RA cured at 60 °C compared with its corresponding specimens at 25 °C. Similarly, Lamprise et al. [99] studied the effects of using high-temperature curing on the increment of the compressive strength of silt geopolymerization made from aggregate and waste washing plant. The results demonstrated positive impacts from increasing the temperature from ambient to 60 °C for three days. The results were also confirmed by a denser matrix and an increase in the density of the specimens. In addition, early high strength was obtained when fly ash-based binders containing RA were subjected to hot curing, while a longer curing time had less influence on further strength development of the matrix [100]. This has also been observed on fly ash-based alkali-activated concretes containing NA, where around 70% and 98% of the 28-day compressive strength was achieved on the third and seventh day, respectively [101].

Table 1 summarizes the proposed solution to improve the mechanical strength and shrinkage of alkali-activated concrete containing RA.

### 2.3. Durability Properties

Durability assessments evaluate the resistance of concretes to various harsh conditions [103]. The causes of concrete deterioration can be loosely grouped into five categories, where the first two are physical, and the other three are chemical [104]:

1. Surface wear due to abrasion, erosion, or cavitation
2. Cracking due to gradients in temperature and humidity, crystallization of salts, or exposure to extremely high or low temperatures
3. Hydrolysis of the binder with soft water
4. Cation exchange between paste and fluids
5. The formation of expansive products due to sulfate attack, alkali-aggregate reaction, or steel reinforcement corrosion

In general, the durability properties of OPC concrete are adversely affected by the incorporation of RA because of greater porosity [105,106,107,108]. In alkali-activated binders; however, the usability of RA might be more suitable because of the higher alkali environment of the matrix [109].

#### 2.3.1. Water Absorption, Sorptivity, and Voids

The major problem with using RA is its greater porosity and high levels of water absorption: 3% and 13% for low-strength and high-strength mortars, respectively [17,36,110]. To mitigate the problems of high levels of water absorption, the moisture level of RA should be approximately 80% [42], and a staged mixing approach can be used (i.e., the addition of extra water to prevent ITZ water flow to RA) [111]. Kathirvel and Kaliyaperumal [69] used pre-wetted and saturated RA in steel-reinforced sodium silicate and hydroxide-activated blast furnace slag concrete. It was revealed that using RA in alkali-activated concretes had a greater impact on the reduction of water absorption, sorptivity, and voids when compared to the use of RA in cementitious compositions [69]. This enhancement could be due to internal curing impacts and the densification of the matrix. Therefore, the minimum water absorption, sorptivity, and voids were found in compositions with 25% saturated RA. Above this content, as shown in Figure 3b and Figure 6, the release of water from the saturated RA to the matrix led to activator dilution and a reduction of pH and, consequently, lower strength development and higher porosity obtained for the compositions. Water absorption and voids were between approximately 4% and 8–10%, respectively [69].

Particle size and the amount of RA affect the porosity and permeability of compositions. Posi et al. [98] indicated that increasing RA/ash ratio and decreasing the proportion of fine aggregates increased water absorption from 10% up to approximately 32%. Fine aggregates filled the gaps and reduced the porosity and water absorption. Interestingly, Hu et al. [112] reported that there are excellent correlations among the water absorption, sorptivity, and volume of permeable voids in alkali-activated fly ash/GGBFS concrete with RA.

Aside from the physical and mechanical properties of RA, the use of different additives has greatly impacted durability performance. When metakaolin was added to high FA-based alkali-activated RA concrete, the porosity, sorptivity, and water absorption decreased by up to 38%, 33%, and 30%, respectively [113]. This was explained by the increase in the amount of denser C-A-S-H gel (as compared to C-S-H gel). Furthermore, it has been reported that the addition of 1% nano-SiO_2_ further decreased porosity, sorptivity, and water absorption.

Accelerated carbonation causes coarser capillary networks in RA. Mastali et al. [78] obtained water absorption of approximately 10% for fly ash based alkaline mortars containing RA. When C-S-H is carbonated, its Ca/Si ratio drops, and it becomes highly porous.

#### 2.3.2. Chloride and Sulfate Diffusion

Chloride diffusion into concrete is a concern due to the corrosion of steel reinforcements, and no formation of detrimental solid phases should take place [114,115,116]. Consequently, these durability indices become significant in reinforced concrete (RC) structures. According to ACI 318–11, concrete ingredients should not contain more than the specified values of water-soluble chloride during the service life [117].

The use of RA (which are not chloride-contaminated) could either increase or decrease chloride diffusion, depending on the chemical reactions with unhydrated tricalcium aluminate (C_3_A). The chemical reaction of chloride ions with unhydrated C_3_A leads to the formation of calcium chloroaluminate hydrate or Friedel’s salt. The chemical products formed in this way reduce the porosity of RA and, consequently, leads to chloride transport slowdowns in concrete containing RA [118,119]. In contrast, the chemical reaction of chloride ions with unhydrated C_3_A causes damage to concrete due to crystallization pressure, which comes from sulfate salt precipitation (e.g., gypsum, ettringite, or thaumasite) [115]. It has been reported that using 25–75 wt % RA in alkali-activated slag concrete resulted in a shallower chloride penetration depth (<≈14 mm) than with OPC concrete prepared with NA [69]. The chemical reactions of chloride ions with the remaining unhydrated C_3_A in RA formed calcium chloroaluminate hydrate and reduced the porosity of alkali-activated slag concrete, while the formation of extra calcium chloroaluminate hydrate in OPC concrete caused crystallization pressure, imposing internal stresses, and subsequently, microcracks formed. Therefore, the chloride diffusion coefficient increased markedly when the amount of RA was increased from 50 wt % to 75 wt % or even more [69]. A similar chloride penetration depth was also reported in high-calcium fly ash geopolymer concretes containing RA or NA [113]. This finding was supported by the formation of Friedel’s salt and the reduced porosity of RA, leading to the retardation of chloride permeability.

Moreover, it was proved by [69] that different sulfuric environments cause different damage levels in compositions containing RA. For instance, in alkali-activated slag concrete containing RA recovered from crushing old OPC concretes, resistance against different sulfate attacks was evaluated, and exposure to MgSO_4_ resulted in consistently greater weight reduction (up to approximately 6%) than with Na_2_SO_4_ (up to approximately 3%) [69]. Under both sulfate attacks, as the amount of RA increased, weight loss due to sulfate exposure increased [69].

#### 2.3.3. Acid Resistance

Under acid attack, the components of OPC concrete dissolve and decompose, so its negative impacts are most pronounced by dissolving calcium hydroxide and decomposing the hydrated silicate and aluminum phases [120]. This phenomenon quickly degrades the strength. Low calcium-based alkali-activated binders or geopolymers are, in general, more acid-resistant than OPC concrete because, in many cases, water absorption and calcium content are lower [121].

The inclusion of unhydrated C_3_A in RA (with a source of calcium) may make alkali-activated concretes more vulnerable to damage from acid compared to the use of NA.

Vavro et al. [122] noticed that resistance to 3% chloric acid strongly depended on the type and amount of RA in alkali-activated blast furnace slag binders. Interestingly, using RA that consisted of 100 wt % recycled bricks increased the compressive strength of more than 20% during acid exposure. When less RA was replaced, or different types of RA (recycled concrete, waste graywacke, waste sand from kaolin washing) were used, the strength decreased by up to approximately 20%.

When high-calcium fly ash alkali-activated binders containing RA were exposed to 3% sulfuric acid, samples could withstand 28 days without significant degradation [28,113]. After 56 days, samples with RA exhibited 20–30% weight loss [28,113].

#### 2.3.4. Freeze and Thaw Resistance

Cyclic freezing and thawing cause the most common cracking and spalling in concrete [123]. The cause of damage is the expansive pressure (9% of volume expansion) from freezing water. However, in addition to hydraulic pressure, osmotic pressure can also exist due to the concentration gradients in capillaries (salt solutions freeze at lower temperatures than pure water) [123].

Similar to other durability properties, the type and content of RA have major effects on the performance of alkali-activated concretes under freeze and thaw resistance. Vavro et al. [124] used crushed clay bricks, and OPC concretes as the RA in slag-based concretes activated by the combination of sodium hydroxide and sodium silicate. It was revealed that after experiencing 100 freeze and thaw cycles, the loss of mass in the composition containing RA from crushed old OPC concrete was three times less than the composition with RA from crushed clay bricks.

Table 2 lists some of the proposed solutions for minimizing the problems of using RA in alkali-activated concretes.

## 3. Toxicity of RA in Alkali-Activated Binders

In the case of using recycled materials, the main environmental problem is the release of heavy metals into the environment through leaching [125]. The leaching out of elements, such as arsenic (As), chromium (Cr), lead (Pb), cadmium (Cd), barium (Br), silver (Ag), and selenium (Se) in alkali-activated fly ash-based concretes causes great environmental concern due to their toxicity and high mobility in the alkaline pH range. This problem becomes more significant in the pore solution of concrete. The addition of calcium results in the formation of additional C-S-H gel, which reduces the porosity, and as a result, it reduces leaching in high pH environments [53]. Therefore, using RA (prepared from old OPC concretes) as a source of calcium with unhydrated C_3_A can reduce the release of hazardous heavy metals [53].

Based on the original sources of RA, these aggregates could release different heavy metals through leaching. Arenas et al. [76] used the EN 12457-4 test to assess the leachate of RA (with a source of calcium). The limits stated in German standards were also used for RA. It was found that the leached contents of the RA were lower than the allowable limits (defined metal concentrations for RA provided from C&D wastes in EN 12457-4, where the allowable limits [in μg/L] are As < 50, Pb < 100, Cd < 5, Cr < 100, Cu < 200, Ni < 100, and Zn < 400) [76].

It was recommended by [126] that prior to the use of RA in compositions, the leaching out of elements of the RA should be assessed based on the chemical sources of RA provided from C&D wastes. Panizza et al. [126] investigated the chemical reactivity of RA provided from old concrete and fired clay through the analysis of leachates obtained after a 24 h leaching test in a 10 M KOH alkali solution. The concentrations of Si and Al were measured and showed that both elements were active in the alkali solution. The leached Al and Si contents recovered from fired clay were more than three times higher, and 50% higher, respectively, than RA, obtained from old OPC concrete. Achtemichuk et al. [127] proposed that saturating RA in water releases the alkalis. They used a simplified approach to assess the leaching content of Na_2_Oe contributed from composition with RA during 28 days, and it was found that 0.08% of RA mass was released during 28 days soaking in water [127].

## 4. Environmental Analysis

The costs and environmental aspects of all cement and cementless-based compositions for construction depending on the sources and the transportation distance of raw materials. The CO_2_ footprint caused by aggregate transportation could reach 20% of total CO_2_ emissions, but this value is significantly governed by the amount of aggregate and distance to local sources [128]. Regarding the use of a high ratio of aggregates to other raw materials in concretes, the transportation of aggregates also has a major effect on the final cost of the compositions. Therefore, using recycled C&D waste as aggregates in cementitious concretes and alkali-activated concretes could have superior impacts in terms of financial and environmental aspects.

In terms of environmental analysis, Yang et al. [128] found that the contribution of the binder results in greater CO_2_ emissions with OPC concrete, while the contribution of aggregate transportation is more critical in alkali-activated binders [129]. In terms of cost analysis, the type of aggregate has the greatest impact on increasing the total cost of fly ash-based alkali-activated concretes [96], so that replacing RA with NA increased the material costs in the range of 30–35% [88].

**Figure 8:**
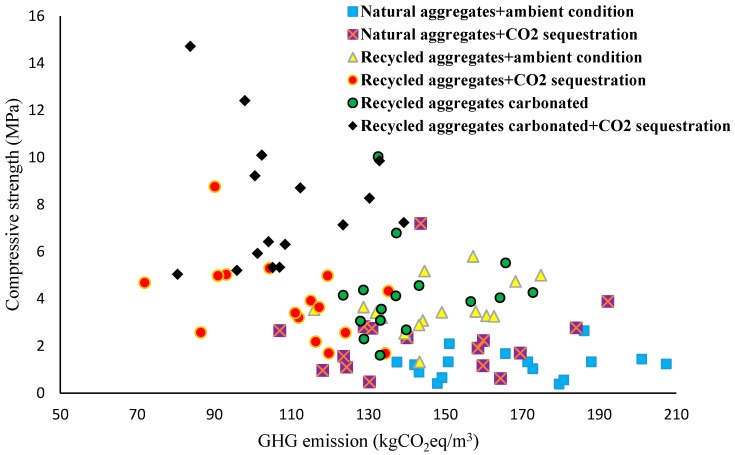
Impacts of different curing regimes (ambient conditions or CO_2_ sequestration) on the compressive strength versus GHG emissions of different fly ash-based alkali-activated concretes containing NA or RA [96].

In 2012, van Deventer et al. [130] proposed that E-Crete (Zeobond’s proprietary geopolymer technology product, which consists of fly ash, slag, and NA) could release emissions equal to 100 to 300 CO_2_ per tonne, while this emission varied in the range of 300 to 900 CO_2_ per ton with OPC content. Yang et al. [128] confirmed that the emission of carbon dioxide into the atmosphere could vary from 100–200 CO_2_ per tonne in different alkali-activated binders (slag, fly ash, and metakaolin) with NA and medium compressive strength (up to 40 MPa). Mastali et al. [96] also reported that GHG emissions in different fly ash-based alkali-activated binders containing RA (as uncarbonated and carbonated) and cured in either a carbonation chamber (named as accelerated carbon dioxide sequestration) or ambient conditions varied in the range of 70 to 200 CO_2_ per ton. Mastali et al. [96] also investigated the influences of different types and contents (aggregate to binder ratios of 4 and 5) of RA under different curing regimes in different fly ash-based alkali-activated concretes. Their results with regard to the compressive strength of the different developed fly ash-based alkali-activated binders versus GHG emissions are shown in Figure 8.

According to the results, regardless of the curing conditions, using RA leads to reduced GHG emissions [96]. Curing specimens with accelerated carbon dioxide sequestration significantly reduced the carbon emissions as compared to cure in ambient conditions. This reduction in GHG emissions could be related to an increase in carbon dioxide uptake [96].

It could be generally concluded that the cure regime has a major impact on the reduction of GHG emissions, and RA treatment using carbonated aggregates could have the greatest impact on decreasing it. A comparison of the results indicates that simultaneous flow-through CO_2_ curing and RA carbonation could decrease GHG emissions by roughly 50% when compared to the use of NA and curing in ambient conditions.

The results, shown in Figure 8, clearly demonstrate that using carbonated RA and flow-through CO_2_ curing reduces GHG emissions and increases strength in alkali-activated concretes, so that GHG emissions of alkali activated concretes varied in the range of 80 to 150 CO_2_ per ton and the compressive strength range from 5 to 15 MPa. The aim of that investigation was the development of bricks with alkali-activated binders containing RA with very low GHG emissions. Because the required strength of a brick is in the range of 5 MPa [129], based on the measured strength, the developed construction materials could be used as footpaths, driveways, and bricks with low GHG emissions.

## 5. Conclusions

This paper reviewed the effects of the use of C&D waste as RA on fresh and hardened state properties of alkali-activated concretes. This review reflects the considerable amount of research that has been conducted, although some aspects are still unclear and need to be further investigated. In this regard, this study has yielded the following remarks.

The main elements affecting fresh state properties when using RA could be due to the highly porous nature and subsequent high levels of absorption of liquid phases of the system by RA as well as the availability of reactive precursors in RA, such as calcium. In order to minimize high levels of water absorption with RA, the two approaches that are used are (1) pre-saturation and (2) the addition of more water during the mixing procedure. Using both these techniques leads to reduced pH values, activator dilution, and compensation for the high levels of water absorption with RA. In addition, releasing water from the pre-saturated RA to the matrix could provide internal curing, although the size of RA has a great impact on the efficiency of internal curing conditions and water reservoir in alkali-activated binders. The setting time of alkali-activated concretes containing RA depends on the dominant gel, where the formation of C-A-S-H gel shortens the setting time.

In general, using RA degrades the mechanical properties of alkali-activated compositions, which leads to the main factors that should be taken into account: (1) RA is low-strength due to the porous and cracked structure; (2) extra water use to minimize high levels of water absorption by RA; and (3) weakness at the ITZ. However, some enhancements in mechanical properties have also been reported, which are caused by (1) the internal curing action and (2) the formation of a denser and stronger ITZ between aggregates and the matrix due partially to the contribution of the unreacted calcium content of the attached cementitious mortars and modification of the matrix microstructure.

The quality and strength of RA and the properties of alkaline solutions were indicated as the most important parameters for the bond properties at the ITZ in alkali-activated concretes. Moreover, the role of the ITZ in crack propagation of concretes containing RA is more critical than with NA.

In general, the treatment methods used with RA to remove adhered mortar from aggregates are either by dissolving it in a solvent or by applying internal stress at the ITZ. Some treatment techniques not only remove the adhered mortar from aggregates but also result in an improvement of the quality of RA through the densification of the microstructure.

The use of pre-saturated RA can minimize shrinkage, water absorption, sorptivity, and voids. Moreover, the type and content of RA significantly affect the performance of alkali-activated concretes under harsh conditions.

OPC has a greater contribution to CO_2_ emissions in cementitious concretes, while the transportation of aggregates is more critical in alkali-activated binders. Therefore, using RA on a construction site eliminates aggregate transportation and brings significant environmental benefits.

Finally, the curing regime has a major impact on the reduction of GHG emissions. Using simultaneous flow-through CO_2_ curing and RA carbonation could decrease roughly GHG emissions by roughly 50% when compared to the use of NA and curing in ambient conditions.

## Figures and Tables

**Table 1:** Summary of the proposed solution to enhance the mechanical characterizations and shrinkage of alkali-activated concretes incorporating RA.

Hardened State Properties	Proposed Solution	References
**Mechanical strength**	Using less than 50% RA (25 to 40 wt %)	[30,36,37,38,39,62,63,64,65,66,67,69]
	Adding a slightly larger quantity of cement (~5%) in the cementitious compositions	[2]
	The improvement at the ITZ by focusing on the quality and strength of RA and the properties of alkaline solutions	[35,68,72]
	Adjustment of water demand of RA with using: (1) pre-saturation and (2) the addition of more water during the mixing procedure	[22]
	Using thermal curing conditions with a temperature of 60–70 °C	[58,97,99,101]
	Increasing the molarity of sodium hydroxide up to a certain point	[28,76,78]
	Using carbon dioxide sequestration as a treatment approach for RA	[95,96]
**Shrinkage**	The internal curing action using RA with a diameter less than 2 mm	[37,82]
	Replacing RA up to 30% (wt %)	[102]

**Table 2:** Summary of proposed solutions to mitigate durability problems when using RA in alkali-activated concretes.

Durability Index	Proposed Solutions	References
**Water absorption, porosity, sorptivity**	The moisture state of RA should be approximately 80% or pre-saturated	[42,69]
	Staged mixing approach could be used	[111]
	Limit RA content	[98]
	Use small size RA	[98]
	Use reactive additive to enhance binder properties	[113]
**Chloride and sulfate diffusion**	Use of 25–75 wt % of RA	[69]
	Increasing sodium silicate to sodium hydroxide ratio	[72]
**Acid resistance**	Using recycled bricks instead of crushed old OPC concretes as RA	[122]
	Use reactive additive to enhance binder properties	[113]
**Freeze and thaw resistance**	Using crushed old OPC concretes instead of recycled bricks as RA	[124]
